# The Many Faces of Amphipathic Helices

**DOI:** 10.3390/biom8030045

**Published:** 2018-07-05

**Authors:** Manuel Giménez-Andrés, Alenka Čopič, Bruno Antonny

**Affiliations:** 1 Institut Jacques Monod, CNRS, UMR 7592, Université Paris Diderot, Sorbonne Paris Cité, 75013 Paris, France; manuel.gimenezandres@ijm.fr; 2 Université Paris-Sud, Université Paris-Saclay, 91405 Orsay, France; 3 Université Côte d’Azur, CNRS, IPMC, 06560 Valbonne, France

**Keywords:** amphipathic helix, membrane deformation, membrane curvature sensor, ALPS motif, phosphatidic acid, lipid packing defect, perilipin, LEA protein, membrane targeting, desiccation

## Abstract

Amphipathic helices (AHs), a secondary feature found in many proteins, are defined by their structure and by the segregation of hydrophobic and polar residues between two faces of the helix. This segregation allows AHs to adsorb at polar–apolar interfaces such as the lipid surfaces of cellular organelles. Using various examples, we discuss here how variations within this general scheme impart membrane-interacting AHs with different interfacial properties. Among the key parameters are: (i) the size of hydrophobic residues and their density per helical turn; (ii) the nature, the charge, and the distribution of polar residues; and (iii) the length of the AH. Depending on how these parameters are tuned, AHs can deform lipid bilayers, sense membrane curvature, recognize specific lipids, coat lipid droplets, or protect membranes from stress. Via these diverse mechanisms, AHs play important roles in many cellular processes.

## 1. Introduction

Amphipathic helices (AHs) are protein sequences that fold into a helical structure upon contact with a polar/non-polar interface. They can be found in many stably folded proteins. However, in this review we will focus solely on AHs that fold in contact with the surface of a bilayer-bound organelle or a lipid droplet inside the cell [1,2]. In such sequences, hydrophobic amino-acids (aa) are regularly distributed every N + 3 and/or N + 4 positions with polar residues in between, thereby allowing the helix to present two faces with opposite chemical features: a hydrophobic face and a polar face. Owing to this segregation, the helix lays down parallel to the membrane interface and anchors the protein in a reversible manner.

Since the development of pioneering studies on amphipathic helical regions in apolipoproteins and secreted antimicrobial peptides [2,3,4], the field of AHs has flourished, with the discovery that many eukaryotic, viral, or bacterial proteins contain AHs which contribute to intracellular membrane targeting, sometimes in combination with other modes of protein–membrane interaction [2,5]. Furthermore, studies have revealed that many AHs act not only as membrane anchors but also fulfill other functions owing to their extended contact with membrane interfaces. These include sensing membrane curvature and the level of lipid unsaturation [6], remodeling membranes into tubular or spherical intermediates [7,8], or acting as a shield to protect membranes or lipid droplets [9,10].

**Figure 1:**
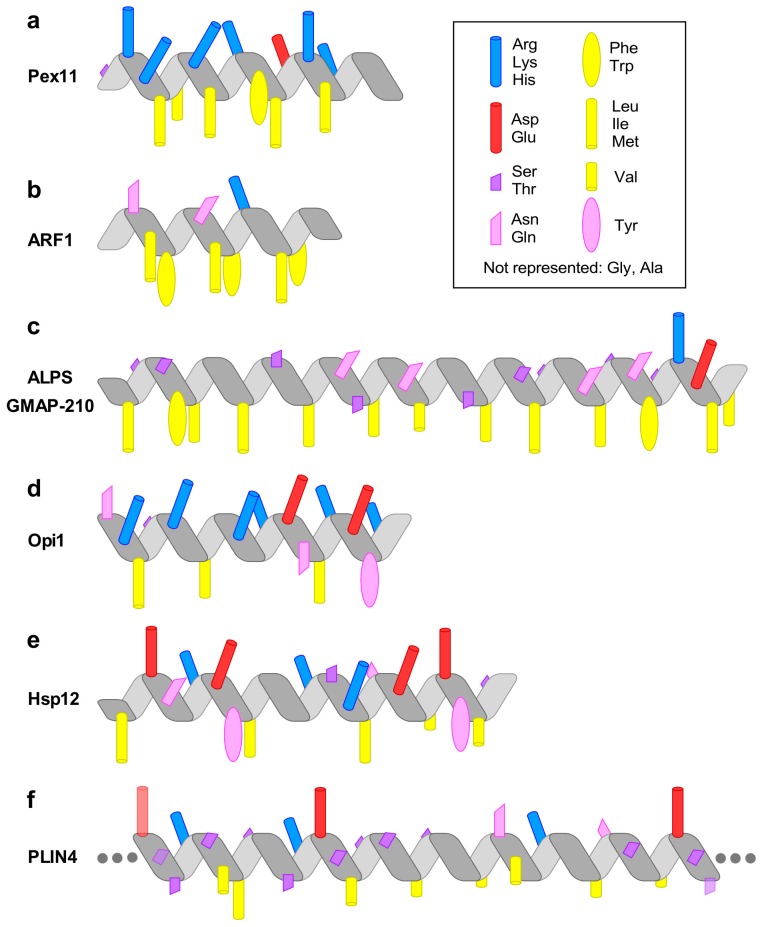
The chemical diversity of amphipathic helices (AHs). The diagrams highlight the most prominent chemical features of the AHs discussed in the text. (**a**) The AH of Pex11 has a highly basic polar face and a prominent hydrophobic face (amino-acids (aa) 66–83 of *Penicillium chrysogenum* Pex11). (**b**) The AH of the small G protein ARF1 contains two bulky hydrophobic residues per helical turn (aa 2–14, human protein). (**c**) The amphipathic lipid packing sensor (ALPS) motif of the golgin GMAP-210 contains one bulky hydrophobic residue per helical turn and is rich in Ser, Thr, and Gly in its polar face (aa 1–38, human protein). (**d**) The AH of Opi1 contains basic residues in its polar face, which have been proposed to bind preferentially to the negatively charged phosphatidic acid (PA) (aa 111–128, *Saccharomyces cerevisiae* protein). (**e**) The four AHs of heat-shock protein-12 (Hsp12) contain both positively and negatively charged residues. The positively charged residues form two wings at the polar/non-polar interface, whereas the negatively charged residues are concentrated in the center of the polar face. The longest helix, helix 4 (aa 74–97 in the *S. cerevisiae* protein), is shown. (**f**) Perilipin 4 (PLIN4) contains a giant and highly repetitive AH of about 1000 aa. The drawing schematizes the chemistry of a single human 33-mer repeat. Large hydrophobic residues are absent from this AH. Instead, the hydrophobic face is rich in Ala, Val, and Thr residues. The polar face contains both positively and negatively charged residues, with the positive charge concentrated on one side of the AH.

**Figure 2:**
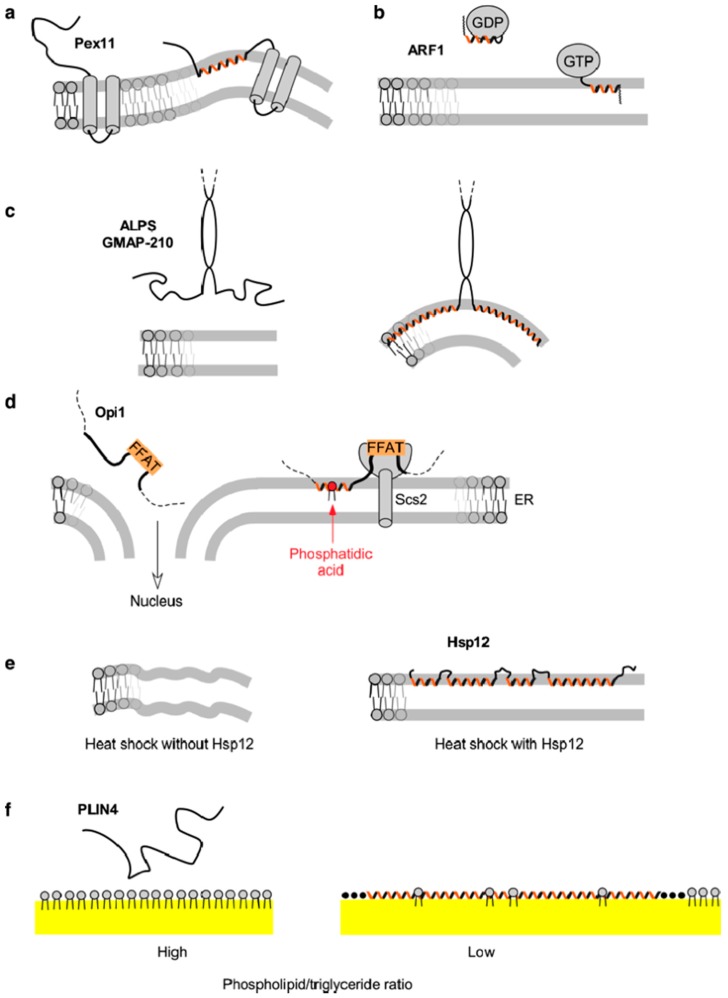
Functional diversity of AHs. This figure illustrates the cellular roles of the AHs shown in Figure 1. (**a**) Membrane deformation induced by the AH of the peroxisomal membrane protein Pex11. (**b**) Guanosine diphosphate/guanosine triphosphate (GDP/GTP) exchange in ARF1 controls the exposure of its AH and thereby the translocation of this small G protein to lipid membranes. (**c**) The ALPS motif of the golgin GMAP-210 captures small vesicles on the basis of their high curvature. (**d**) The yeast transcriptional repressor Opi1 is retained at the endoplasmic reticulum (ER) membrane through its dual interaction with PA and the ER receptor Scs2 (member of the VAP protein family), via an AH and a FFAT motif, respectively. When the amount of PA decreases, Opi1 is released from the ER and is translocated to the nucleus where it represses genes involved in lipid synthesis. (**e**) The adsorption of the large AH region of the heat shock protein Hsp12 has a protective effect on the plasma membrane by adjusting its physical properties. (**f**) The giant AH of PLIN4 coats lipid droplets under conditions of insufficient phospholipids by directly substituting the phospholipid monolayer.

In this review, we use a few examples of membrane-adsorbing AHs to illustrate how variations in length and amino-acid sequence (Figure 1) provide AHs with different interfacial properties and, thereby, different cellular functions (Figure 2). This is because the repertoire of hydrophobic and polar residues is vast enough to allow the synthesis of helices that, apart from their amphipathic character, differ considerably in their possible interfacial interactions. To facilitate their comparison, the AHs are represented with a normalized scheme that aims to highlight their distinguishing features (Figure 1). However, it should be noted that neither the structure of these AHs nor their position at the lipid interface is known in most cases. Thus, these drawings should be taken as working models, not as definitive pictures.

## 2. Predictions and Experimental Approaches to Study Amphipathic Helices

The analysis of AHs is facilitated by bioinformatics tools such as Heliquest [11] which project any amino-acid sequence onto a helical wheel and calculate various parameters such as AH hydrophobicity, charge, and hydrophobic moment (an index of the amphipathic character of the putative helix). Note that some AHs slightly depart from the archetypal α-helical structure, which contains 3.6 aa per turn. These so-called 3–11 helices contain 3.67 aa/turn (giving an integer number of 11 aa for three turns) and are generally associated with extended sequences made of repetitions of 11, 22, or 33 aa present in apolipoproteins [4], synucleins [12], and perilipins, respectively [10].

Even though the bioinformatics approach is helpful in order to identify, characterize, and mutagenize some putative AHs, it does not provide proof of AH formation. Furthermore, the AH structure requires an appropriate but poorly defined amino-acid sequence interacting with a hydrophobic interface. Many membrane-interacting AH sequences have a low probability of α-helical formation and are in fact intrinsically unstructured in solution owing to an abundance of Gly, which increases conformational freedom. Only when facing an appropriate interface do they adsorb and fold into a helix [1,2].

The structures of membrane-binding AHs are generally less well-established than those of helices in stably-folded protein domains because of the difficulty in crystallizing proteins in an interfacial environment. However, a combination of site-directed mutagenesis and spectroscopic and biochemical methods can give reasonable clues about the relevance of a predicted AH (for a typical example, see [13]). Among these methods are: (i) liposome–protein binding assays, in which the fraction of bound protein is recovered by flotation or sedimentation; (ii) circular dichroism (CD) spectroscopy to assess the ability of the sequence of interest to fold into a helical structure; and (iii) various fluorescence methods to probe the environment of selected intrinsic residues (e.g., tryptophan) or extrinsic probes such as 7-nitrobenz-2-oxa-1,3-diazol-4-yl (NBD). Binding assays at the level of single liposomes represent a more sophisticated type of analysis [14].

Deeper structural analysis of AHs is sometimes performed but requires more advanced approaches. Various nuclear magnetic resonance (NMR) methods allow for the visualization of the structure and aa environment of AHs in membrane mimetic systems such as bicelles, which are discoidal lipid bilayer patches [15], or, more classically, in detergent (e.g., sodium dodecyl sulfate—SDS) micelles [16]. However, the small size of micelles can lead to helix breakage (discussed in [17]). X-ray diffraction in the presence of model bilayer membranes gives information about the time averaged density of AH atoms across the bilayer normal [18]. In the case of the apolipoprotein A-I, this method indicates that the axis of the helix is positioned at the level of the glycerol atoms, almost exactly between the polar and non-polar region of the bilayer, whereas large hydrophobic residues insert as far as the middle of the lipid acyl chains. Finally, in site-directed spin labeling, selected aa along the sequence are mutated into Cys, which are then labelled with spin probes to give information about the position of aa relative to the membrane surface by electron paramagnetic resonance spectroscopy [9,17]. A most spectacular example is the structural characterization of α-synuclein, in which aa from positions 25 to 90 was sequentially labeled, leading to a high-resolution reconstruction of a continuous helix that extends parallel to a phospholipid membrane with a 3–11 helical periodicity [17].

These various biophysical methods give details about the depth of insertion of the AH into the membrane, which can be decisive for understanding its impact on membrane organization (e.g., curvature) [16,17,18]. However, one difficulty in structural studies is finding a model membrane system that is simple enough to be compatible with physical measurements, and yet similar enough to an authentic membrane. Due to their small size and highly charged surface, SDS micelles can lead to artefactual protein conformations (as discussed in [17]). As illustrated by the few examples discussed below, much information stems from experiments on liposomes of defined size and lipid composition; however, inappropriate liposome composition or concentration can likewise produce experimental artefacts.

Molecular dynamics simulations can give some plausible mechanisms for how the AH sequence adsorbs and folds at an interface. In the few examples that have been studied so far, a few hydrophobic residues start inserting into the membrane in a random manner, followed by a gradual increase in AH folding and by the coalescence of the cavities hosting the hydrophobic residues [19,20,21]. Therefore, the final footprint of the helix in the membrane is much larger than the lipid packing defects initially present at the interface. However, AH folding at membrane interfaces is a slow process at the scale of molecular dynamics and has been poorly characterized so far.

## 3. Pex11 and Membrane Deformation

Pex11 is a conserved peroxisomal protein whose expression level modulates the number of peroxisomes in a cell [22]. It induces tubulation of peroxisomal membranes, which is followed by a fission step performed by the dynamin-related GTPase Dnm1 [23]. Pex11 contains an AH in its N-terminal part, followed by two predicted transmembrane helices (Figure 1a and Figure 2a). The AH has a well-developed hydrophobic face and a polar face rich in positively charged aa (Figure 1a). In vitro, it binds significantly better to liposomes containing negatively charged lipids that mimic the peroxisomal membrane as compared to neutral phosphatidylcholine (PC) liposomes [24]. Mutational analysis indicates that both hydrophobic and electrostatic interactions contribute to this binding. Amphipathic helices peptides from diverse yeast species as well as from the human Pex11 orthologue induce strong tubulation of negatively charged liposomes [24]. Similarly, tubulation of peroxisomes in the yeast *Hansenula polymorpha* lacking Dnm1 depends on Pex11 AH [24], and there is a similar requirement for Pex11β in human cells [25]. However, in addition to the amphipathic character of Pex11 AH, oligomerization may also contribute to its tubulation activity [25,26,27].

There are many proteins that shape membranes by a mechanism similar to that described for Pex11. In general, their AH is relatively short, has a strong hydrophobic moment, and acts in concert with other structural elements that position the helix close to the membrane surface. The large GTPase atlastin, which mediates endoplasmic reticulum (ER) membrane fusion, contains two transmembrane segments flanked by the GTPase domain and by an AH on the N-terminal and C-terminal ends, respectively. The AH hydrophobic face is rich in aromatic aa, whereas the polar face contains three basic and three acidic residues, with a resulting high hydrophobic moment [28]. This AH participates in ER membrane fusion by destabilizing the lipid bilayer. Although the mechanism is still uncertain, it was proposed that the AH insertion causes the displacement of the negatively charged phospholipid heads, leading to exposure of their hydrocarbon chains and bilayer destabilization. Facilitation of fusion also occurs when the AH is separated from the rest of atlastin, but in that case a 50-fold higher AH concentration is required, highlighting the advantage of having the AH within the same protein chain [28].

A recent publication demonstrates that a similar mechanism is involved in mitochondrial fusion, specifically the fusion of outer mitochondrial membranes that is mediated by mitofusins, large GTPases similar to atlastin [29]. Like in the case of atlastin, mitofusin-mediated mitochondrial fusion requires a conserved AH adjacent to the transmembrane domains that dock mitofusin in the mitochondrial membrane. This AH is similar in character to that of atlastin, although it contains even higher amounts of charged residues and no aromatic residues. Notably, it lacks any Gly residues and accordingly is quite helical even in the absence of membranes. The authors propose that, like in atlastin, this AH acts to destabilize the mitochondrial bilayer. Interestingly, a chimeric mitofusin containing the transmembrane domains of atlastin is targeted to the ER instead of the mitochondria and can at least partially restore the ER morphology in yeast lacking the atlastin orthologue [30]. This group identified another AH on the other side of the mitofusin transmembrane domains, but the role of this segment is less clear. Given the rather different lipid compositions of ER and mitochondrial membranes, it will be interesting to see to what extent the functions of these two large GTPases, and in particular their AHs, overlap.

Endocytosis is one of the best characterized mechanisms of membrane deformation in vivo and in vitro. Central to membrane deformation in endocytosis are BAR domain-containing proteins, which through their banana shape and positively concave face interact electrostatically with the lipid bilayer and deform it. Some of these BAR domains are flanked by AHs. Amphipathic helices are also present in other endocytic proteins such as epsin. The relative contribution of BAR domains and AHs in membrane binding, deformation, and fission is a matter of debate [7,8,31,32]. More recently, protein crowding has been evoked as another mechanism of membrane deformation [33,34]. In the crowding regime, an AH may act as a membrane anchor but is not required for destabilizing the bilayer; for example, Stachowiak et al. demonstrated that an epsin mutant whose AH was replaced by a His-tag was also sufficient to deform liposomes [33]. Careful work will be needed to determine how these different effects combine in the complex cellular environment.

## 4. ARF1—A Small Amphipathic Helix Regulated by a GDP/GTP Switch

The small G protein ARF1 is involved in intracellular vesicle trafficking [35]. It is myristoylated on its N-terminal Gly, followed by a small AH of 14 a.a. The hydrophobic face of the AH is highly developed, with one pair of strong hydrophobic residues per turn (Leu–Phe or Ile–Phe) (Figure 1b). When ARF1 is in the guanosine diphosphate (GDP) state, the AH is folded, with the hydrophobic residues pointing towards the protein core. Upon activation by a nucleotide exchange factor, which promotes the replacement of GDP by guanosine triphosphate (GTP), the AH can no longer interact with the protein core and instead extends on membrane surface [15]. Thus, GDP/GTP exchange controls ARF1 interaction with lipid membranes (Figure 2b). The interaction of ARF1–GTP with model membranes is very strong, with a spontaneous dissociation rate in the order of minutes. When hydrophobic residues of the AH are mutated to Ala, the membrane interaction of ARF1–GTP is reduced [34]. Importantly, ARF1–GTP can bind to most interfaces including lipid bilayers of different curvature and lipid composition (saturated/unsaturated, charged/neutral) as well as to lipid droplets covered by phospholipids, but not to liquid-ordered domains [36,37,38,39]. In the cell, ARF1 localization is therefore controlled by the distribution of exchange factors, among which many reside at the Golgi [35]. 

The coupling between GDP/GTP switch and AH exposure allows ARF1 to control the membrane recruitment of numerous effectors [35]. In some cases, this process is followed by membrane deformation. Thus, ARF1 and cognate proteins such as SAR1 drive the recruitment of protein coats, which polymerize into a spherical shell to promote vesicle formation. The high surface density of the AHs of ARF1/SAR1 under coat formation contributes, in combination with other factors (i.e., coat structure and protein crowding), to membrane shaping [40,41,42,43,44,45,46].

## 5. The Amphipathic Lipid Packing Sensor Motif: Amphipathic Helices with Sparse Hydrophobic Residues to Sense Membrane Curvature

Amphipathic lipid packing sensors (ALPS) are AHs characterized by a polar face made of small polar residues, notably Ser, Thr, and Gly (Figure 1c). Furthermore, due to a lack of charged residues in the polar face, electrostatic interactions do not participate in the interaction of ALPS motifs with membranes. Instead, membrane adsorption is driven by the hydrophobic effect. Amphipathic lipid packing sensor motifs are very sensitive to both lipid unsaturation and membrane curvature, and therefore bind poorly to most membranes, except those containing a high amount of monounsaturated lipids (e.g., C16:0–C18:1–PC) and a high curvature (radius < 50 nm) [47,48] (Figure 2c). Several mutagenesis studies indicate that the atypical amino-acid composition of ALPS motifs is critical for their dual sensitivity. Thus, mutating an ALPS motif with two Lys close to the polar/non-polar interface causes a loss in the specificity for curved membranes due to electrostatic interactions [6].

A more recent study suggests that another key factor for membrane curvature sensing lies in the sparse distribution of the large hydrophobic residues in the AH [49]. In contrast to the AH of ARF1, which contains two hydrophobic residues per turn, the ALPS motif of the Golgi tether GMAP-210 (Golgi-microtubule-associated protein 210) contains one hydrophobic residues per turn (Figure 1b,c). Condensing the ALPS motif of GMAP-210 to pair up its hydrophobic residues makes the resulting AH more promiscuous: in vitro, it binds to liposomes regardless of their curvature; in the cell, it can no longer specifically recognize small vesicles over flat organelle surfaces, in particular lipid droplets [49]. The AH of GMAP-210 is therefore optimized for trapping small neutral vesicles that transport proteins between the ER and the Golgi apparatus. This selectivity can be demonstrated by introducing synthetic vesicles into living cells: these vesicles accumulate around the Golgi as a function of their physico-chemical properties in a GMAP-210-dependant manner, or can be even targeted to the mitochondria using a mitochondrially-targeted GMAP-210 construct [49].

## 6. Specific Recognition of Lipids by Amphipathic Helices: Opi1 and Other Examples

By being embedded at the interface between the polar and non-polar regions of membranes, AHs necessarily contact many lipids. The question then arises as to whether AH insertion is solely driven by bulk membrane properties (notably lipid packing defects and electrostatics) or whether specific interactions with defined lipid species also contribute to AH binding.

The binding of the yeast transcriptional repressor Opi1 to the ER membrane depends on phosphatidic acid (PA) and on the interaction of Opi1 with the ER protein Scs2, member of the VAP protein family [50,51] (Figure 1d and Figure 2d). In the absence of PA, Opi1 translocates to the nucleus where it represses several genes involved in membrane lipid biogenesis [50,52]. Opi1 senses PA with an AH that has a positively charged polar face rich in Lys [53] (Figure 1d). Depending on its membrane environment, PA displays one or two negative charges [54]. To determine if the binding of Opi1 AH to membranes is driven by electrostatics or by stereospecific interactions, its membrane affinity was tested on liposomes containing increasing concentrations of PA or phosphatidylserine (PS), for example 20% PA or 40% PS to maintain a similar net charge. Interestingly, Opi1 AH interacted more strongly with the PA-containing liposomes, suggesting a stereospecific interaction with PA [53] (Figure 1d). 

Using molecular dynamic simulations, Hofbauer et al. identified two motifs that contributed to the preference of the Opi1 AH for PA versus other negatively charged lipids [53]: one composed of three Lys and the second of Lys-Arg-Lys. Each motif forms a three-finger grip that is able to accommodate the small polar head of PA but not the larger polar head of PS. When all Lys are mutated to Arg, the AH can no longer distinguish between PA and PS.

The AH of the yeast protein Spo20 is also sensitive to PA levels. It has a polar face very rich in basic residues, including three His, displaying a chemistry that is strikingly different from that of Opi1 AH [55]. Whereas Spo20 AH was suggested to specifically recognize PA in membranes, careful in vivo and in vitro analysis revealed that this AH is primarily sensitive to lipid charge, independent of the exact nature of the anionic lipids present [56]. Indeed, once the differences in charge of PA and other negative lipids are corrected, the affinity of Spo20 AH for PA, PS, or phosphatydilinositol 4 phosphate-containing liposomes is remarkably similar [56]. Therefore, the use of Spo20 as a reporter for the quantity of PA in membranes should be taken with caution (for further reading on PA sensing, see [57]). In the future, an artificial helix with several (Lys)3 and Lys–Arg–Lys motifs, inspired by the Opi1 AH, could be developed to a get a more specific fluorescent reporter for PA in cellular membranes.

Cholesterol represents a very different kind of membrane lipid, but one whose concentration in cellular membranes highly varies and is carefully regulated. It can specifically interact with membrane proteins to regulate their function by filling a selective cholesterol-binding pocket [58], but it is less clear whether an AH could specifically contact a cholesterol molecule embedded in a bilayer. Chua et al. recently proposed an intriguing hypothesis for squalene monooxygenase (SM), an enzyme in the biosynthetic pathway of cholesterol located at the ER [59]. In the presence of excess cholesterol, SM is degraded. A small AH of 12 aa is present in the SM sequence, containing several large hydrophobic residues and a poorly developed polar face. A combination of mutagenesis data, molecular dynamics simulations, and CD measurements suggests a feedback model, whereby the AH is embedded in the ER membrane at low cholesterol but becomes displaced and unfolds in the cytosol when cholesterol concentration increases. The authors propose that the AH displacement is caused by the membrane becoming thicker and more condensed due to cholesterol increase, with cholesterol therefore indirectly but specifically regulating AH binding. Together with an upstream disordered region, the unfolded AH then signals through the ubiquitination-proteasome system, inducing SM degradation [59]. However, the mechanism by which the AH of SM senses cholesterol levels awaits further investigation.

In plants, the 140K replication protein of the turnip yellow mosaic virus precisely targets the outer chloroplast membrane during viral replication. The 140K replication protein contains two short AHs, separated by a short loop. Both AHs have a high hydrophobic moment due to a well-developed hydrophobic face containing two aromatic residues, and a polar face that is positively charged. The two AHs have been shown to specifically target the outer chloroplast membrane in vivo [60]. This targeting can occur even when one or the other AH is mutated, suggesting that the two AHs may be at least partially redundant for targeting. Because the outer membrane of the chloroplast contains unique lipids, notably sulfolipids and mono- and di-galactosyldiacylglycerol, further investigations are now needed to assess whether specific interactions may exist between these AHs and these unique lipids, or whether the lipids impart particular bulk properties on the outer chloroplast membrane.

## 7. Amphipathic Helices that Respond to Environmental Changes: Small Heat-Shock Protein Hsp12

Whereas membranes of warm-blooded animals generally do not experience large fluctuations in physical environmental conditions, membranes of microorganisms and plants have to be able to adapt to changes in temperature and humidity. This can happen through changes in membrane lipid composition or accumulation of dissacharides, notably trehalose. Recent work suggests that binding of peripheral proteins may be another important way of changing membrane physical properties by which a variety of cells cope with a varying environment. An interesting example is the small heat shock protein Hsp12 in yeast, which rescues cell growth under various stress conditions by stabilizing the plasma membrane [9]. Like many other heat shock proteins, Hsp12 is present in cells at a low copy number under standard growth conditions but can be induced more than 100-fold by harsh conditions. However, Hsp12 does not appear to interact with other proteins, and is in fact disordered in solution. Instead, it can bind directly to the plasma membrane via four independently-folding non-interacting AHs that represent the majority of its 109 aa sequence, and its binding was shown to increase the stability of model membranes [9,16] (Figure 1e and Figure 2e).

Expression of intrinsically disordered proteins, many of which have been demonstrated to fold into AHs in contact with membranes, is in fact emerging as a widespread mechanism of coping with fluctuations in physical environmental conditions. Late embryogenesis abundant (LEA) proteins represent a large group with members identified in plants and also in some invertebrate animals [61]. Among these, members of the dehydrin family are particularly interesting: these are modular proteins with a very particular amino-acid composition that protect plants against drought and cold. Several of them have been shown to contain a series of short but strong AHs that fold on synthetic membranes, for example dehydrin K2 from *Vitis riparia* (frost grape) [62]. This protein can protect liposomes against fusion during freeze-thaw cycles and can lower membrane phase transition temperature. Another member of the family, Lti30, is found in *Arabidopsis.* The presence of short lipid-induced AHs in Lti30 has been confirmed by NMR, and this protein also reduced lipid phase transition of model membranes [63,64]. Also in *Arabidopsis*, cold-regulated (COR) proteins target and stabilize mitochondrial or chloroplast membranes, possibly via poorly-hydrophobic AH sequences [65,66]. Striking examples from the animal kingdom are LEA proteins from the brine shrimp *Artemia franciscana*, which contain long predicted AHs of more than 100 aa and protect liposomes against desiccation, especially in combination with trehalose [67]. Finally, a number of disordered proteins have been identified in the unicellular tardigrades, which can survive several years of desiccation but produce no or very little trehalose [68].

Much work is still needed to understand how these fascinating proteins modulate membrane properties, leading to possibly very important technological applications.

## 8. Amphipathic Helices Acting as Coats: The (Curious) Case of Perilipin 4

Perilipins are a family of proteins that reversibly associate with lipid droplets and mediate in the regulation of these intracellular organelles [69]. Lipid droplets are unusual in that they contain a hydrophobic core of neutral lipids and a monolayer of phospholipids and proteins [70]. All mammalian perilipins contain a predicted AH region that contributes to their lipid droplet localization [71,72,73]. This region is by far the longest in perilipin-4: the human sequence suggests a continuous AH of more than 950 aa, which, when folded, would measure about 140 nm. Indeed, a purified peptide of 660 aa is unfolded in solution but adopts a highly helical conformation in the presence of a lipid surface [10]. The AH belongs to the family of helices 3–11 and is composed of 33 aa repeats that are remarkable in their degree of conservation and in their lack of large hydrophobic residues (Figure 1f). The low hydrophobicity combined with the high length of this AH is essential for its specificity for lipid droplets in cells. Accordingly, the AH interacts very weakly with bilayer liposomes, but can directly bind to neutral lipids and act as a replacement for the phospholipid monolayer both in vitro and in cells [10] (Figure 2f). Thus, perilipin-4 AH appears optimized for coating lipid droplets and could be important for their stabilization, for example during adipocyte differentiation. A striking feature of this AH is also the distribution of charged residues in the polar face of the AH; their asymmetric organization is not optimal for interacting with a charged lipid surface, but they may instead be mediating lateral inter-helical interactions that would stabilize the protein coat [10] (Figure 1f).

Although perilipins have often been described as lipid droplet coats, it is currently not known whether other members of the family can act in a manner similar to perilipin-4. Interestingly, perilipins share some structural homology with the apolipoproteins [74], whose AHs also interact with neutral lipids to form small lipoprotein particles [75,76]. Conversely, what makes the oil–water interface adapt to some AHs is not well understood, although molecular dynamics simulations indicate a large increase in lipid packing defects under conditions of low phospholipid density [77].

## 9. Conclusions

Except for their amphipathic character, the various AHs that are presented here are very different in their composition (length, amino-acid sequence) and in their surface-binding properties (Figure 1 and Figure 2). Two most contrasting examples are the ARF1 AH and the PLIN4 AH, which differ in all parameters: their length (12 vs. 950 aa), their hydrophobic residues (Leu, Phe vs. Ala, Val, Thr), and their polar residues (uncharged vs. charged) (compare Figure 1b,f). These differences translate into strikingly different binding properties: ARF–GTP binds to most lipid membranes, whereas Plin4 is specific for the lipid droplet surface [10,36,37]. Similarly, the ALPS motif and the AH of α-synuclein display contrasting chemistries and have been shown to recognize different transport vesicles [78]. In most cases, however, we still miss an atomic description of the interaction between an AH and its preferred lipid surface.

However, factors other than the AH–lipid surface interaction can also play a decisive role in regulating AH targeting or function. The presence of transmembrane regions in Pex11 and atlastin or mitofusin necessarily impose their subcellular localization, making the AH a domain important for membrane shaping, but not for targeting. In α-synuclein, the highly acidic region downstream of the AH region exacerbates the sensitivity of the AH to physical membrane parameters [79]. Furthermore, the properties of α-synuclein in vivo are also linked to its interactions with other proteins and to its tendency to self-aggregate into fibrils, a process that is exacerbated by pathological mutations [80]. A different type of example is CTP:phosphocholine cytidylyltransferase (CCTα), which contains one of the most studied AHs so far, but whose exact place of function in the cell has been highly debated [81]. A recent study indicates that, in vivo, this protein resides almost exclusively in the nucleus, suggesting that the inner membrane of the nuclear envelope is the only membrane that CCTα actually senses under most physiological contexts [82]. Lastly, a full understanding of not only giant AHs such as PLIN4, but also apolipoproteins and Hsp12, which necessarily cover very large surfaces, requires a better evaluation of their overall conformation (straight vs. kinked), of their potential intra- and intermolecular interactions, and of their ability to cope with the very crowded environment on the surface of cellular organelles.
